# Types and Frequency of Infusion Pump Alarms: Protocol for a Retrospective Data Analysis

**DOI:** 10.2196/10446

**Published:** 2018-06-14

**Authors:** Kevin R Glover, Rachel R Vitoux, Catherine Schuster, Christopher R Curtin

**Affiliations:** ^1^ Scientific Affairs B. Braun Medical Inc Allentown, PA United States; ^2^ Clinical Consulting Services B. Braun Medical Inc Allentown, PA United States; ^3^ Medical Affairs B. Braun Medical Inc Allentown, PA United States

**Keywords:** medical device, alarms, infusion pumps, alarm fatigue, patient safety

## Abstract

**Background:**

The variety of alarms from all types of medical devices has increased from 6 to 40 in the last three decades, with today’s most critically ill patients experiencing as many as 45 alarms per hour. Alarm fatigue has been identified as a critical safety issue for clinical staff that can lead to potentially dangerous delays or nonresponse to actionable alarms, resulting in serious patient injury and death. To date, most research on medical device alarms has focused on the nonactionable alarms of physiological monitoring devices. While there have been some reports in the literature related to drug library alerts during the infusion pump programing sequence, research related to the types and frequencies of actionable infusion pump alarms remains largely unexplored.

**Objective:**

The objectives of this study protocol are to establish baseline data related to the types and frequency of infusion pump alarms from the B. Braun Outlook 400ES Safety Infusion System with the accompanying DoseTrac Infusion Management Software.

**Methods:**

The most recent consecutive 60-day period of backup hospital data received between April 2014 and February 2017 from 32 United States-based hospitals will be selected for analysis. Microsoft SQL Server (2012 - 11.0.5343.0 X64) will be used to manage the data with unique code written to sort data and perform descriptive analyses. A validated data management methodology will be utilized to clean and analyze the data. Data management procedures will include blinding, cleaning, and review of existing infusion data within the DoseTrac Infusion Management Software databases at each hospital. Patient-identifying data will be removed prior to merging into a dedicated and secure data repository. This pooled data will then be analyzed.

**Results:**

This exploratory study will analyze the aggregate alarm data for each hospital by care area, drug infused, time of day, and day of week, including: overall infusion pump alarm frequency (number of alarms per active infusion), duration of alarms (average, range, median), and type and frequency of alarms distributed by care area.

**Conclusions:**

Infusion pump alarm data collected and analyzed in this study will be used to help establish a baseline of infusion pump alarm types and relative frequencies. Understanding the incidences and characteristics of infusion pump alarms will result in more informed quality improvement recommendations to decrease and/or modify infusion pump alarms, and potentially reduce clinical staff alarm fatigue and improve patient safety.

**Registered Report Identifier:**

RR1-10.2196/10446

## Introduction

### Background

The high incidence of alarms in hospital settings presents a potentially serious problem for patients and caregivers. Between 1983 and 2011, the potential variety of medical device alarms proximate to an intensive care unit (ICU) bed increased from 6 to 40 [1]. Medical devices emitting these alarms include, but are not limited to, physiological monitors, ventilators, pulse oximetry machines, and infusion pumps. It has been reported that these devices share similar alarm volume characteristics that range between 58 and 85 decibels, which compete for clinician attention in patient settings with background noise ranging between 56 and 76 decibels [2,3]. Alarms may interfere with a patient’s sleep, cause unnecessary anxiety, and potentially negatively impact healing [4,5].

Eighty to 99% of medical device alarms are generally thought to be clinically insignificant and/or require no intervention. These nonactionable alarms include those emitted from physiological monitors when parameter thresholds are set too narrowly, causing clinically insignificant alarms to occur [5,6]. It has been suggested that the abundance of nonactionable alarms results in desensitizing clinical staff to all alarms, in turn affecting response time to those that are actionable [5,7,8]. This desensitization, commonly described as alarm fatigue, is an increasingly critical safety issue that can lead to potentially dangerous delays or nonresponse to actionable alarms, which require clinical staff intervention to resolve.

In an effort to reduce patient risk associated with alarm fatigue, alarm management has become a priority issue for national guidance organizations, including The Joint Commission, the Food and Drug Administration, the Association for Advancement in Medical Instrumentation, Emergency Care Research Institute (ECRI), and the American College of Clinical Engineering Healthcare Technology Foundation. In 2016, ECRI listed clinical staff alarm desensitization as a top 10 health technology hazard, stating that fatigue due to the high incidence of nonactionable alarms could result in missed, actionable ventilator alarms that require clinician intervention [9]. The Joint Commission [10] also listed alarm management as a National Patient Safety Goal, stating in 2018 that quality improvements must be made to ensure timely clinical alarm response time. While national guidance organizations have established goals to reduce patient risk through more effective clinical alarm management in general, no research has been conducted to benchmark alarm data to inform specific quality improvement recommendations for infusion pumps specifically [4,8,9,11].

The current evidence related to the incidence of nonactionable and actionable medical device alarms and their associated impact on the desensitization of clinical staff is limited. Research has primarily focused on electrocardiograms (ECGs), physiologic monitors, and pulse oximetry in the telemetry and ICUs, where the total alarm incidences of these devices is thought to be highest [1,5,12]. Alarm incidence in the ICU has been reported as high as 45 times/patient/hour [1], of which 77% are ineffective and/or ignored [13]. Interventions to mitigate nonactionable alarms, including better ECG electrode placement, adjusting alarm thresholds, and education on monitor capabilities and alarms, have resulted in a 12% to 89% reduction in these types of alarms [4,6,7,14-18].

The recommendations to reduce the nonactionable alarms of physiologic, ECG, and pulse oximetry monitors are not applicable to infusion pumps. Most infusion pump alarms are actionable; that is, they continually alarm until addressed by clinical staff. Infusion pump alarms have been found to contribute to 10% to 12% of total ICU alarms with 0% of these alarms being ineffective [1,13]. However, compared to other medical device alarms, infusion pump alarms can last longer [13] and may account for approximately 5% of the infusion time [19]. The differences between high-proportion, nonactionable physiological monitoring alarms and the low-proportion, actionable alarms of infusion pumps require an investigation to study the specific types and frequencies of infusion pump alarms. It is important to note the difference between infusion pump “alarms” and “alerts.” While there has been a concerted effort to aggregate data related to smart pump programing dosing alerts in order to measure impact on drug library compliance [20], the clinical and caregiver impact of postprogramming infusion pump alarms remains largely unexplored. For the purpose of this study, an infusion pump alarm is defined as an audible and visual signal during pump operation that requires the user to address/resolve to silence the alarm.

Before making recommendations to decrease or modify what are believed to be mostly actionable alarms, understanding the incidences and characteristics of infusion pump alarms is necessary. Ultimately, benchmarking the incidences and characteristics of infusion pump alarms will lead to more informed quality improvement recommendations to decrease and/or modify infusion pump alarms while both reducing clinical staff alarm fatigue and maintaining patient safety.

### Objectives

The primary objective of this study is to establish baseline data related to the types and frequency of infusion pump alarms from the B. Braun Outlook 400ES Safety Infusion System with DoseTrac Infusion Management Software. Alarm type, duration, day of week, time of day, drug name, care area, number of deliveries, and number of pumps in use will be captured and evaluated to determine if alarm types and frequency differ according to hospital/unit and, if so, suggest what factors may contribute to these differences.

## Methods

### Study Design

This retrospective study [21] will describe the types and frequency of alarms that occur during infusions with the B. Braun Outlook 400ES Safety Infusion System via DoseTrac at 32 hospitals collectively using approximately 13,000 large volume infusion pumps (April 2014 to February 2017). The DoseTrac application resides on each hospital’s server, and holds up to 18 months of data, with the ability to automatically back-up unlimited amounts of data in a separate file. We will utilize back-up copies of this data previously obtained for analytic services and the most recent consecutive 60-day period will be selected for analysis and summarized using descriptive statistics. The majority of the hospitals (n=28) are located on the East Coast (Pennsylvania, New Jersey, New York, Maryland, Virginia, North Carolina, and Florida); the others (n=4) are located in Kentucky, Iowa, and California.

### Investigational Infusion Device

The B. Braun Outlook 400ES Safety Infusion System is an electrical, external, large volume pump intended primarily for use in hospital, ambulatory, and/or extended care settings. The pump is intended for use with adult, pediatric, and neonatal patients and is equipped with distinct audible and visual alarm signals to indicate Keep Vein Open (KVO), low battery, and other alarm conditions [22]. This pump’s system can customize up to 300 drugs and 15 care areas. The system provides for 2-way wireless communication allowing inbound receipt of drug library files and patient-specific infusion orders, along with outbound transmission of infusion data. The pump also includes a software application which collects, displays, and stores infusion data. The management and analysis of these data allows clinicians to continuously improve the quality and safety of intravenous (IV) infusion protocols. This software application consists of 3 components:

1. A real time infusion data collection service.
2. A database that stores up to 18 months of accumulated infusion data so that it can be displayed or reported in a useful way.
3. A Web application with:
  - Real-time remote views of active infusion pump status that is automatically updated every 5 seconds.
  - Retrospective reports on various infusion metrics.
  - A report scheduler that creates and saves reports/templates and schedules for email delivery [22].

Infusion data is automatically transmitted wirelessly from the pump across the hospital’s secured wireless network and collected by the application for availability to both end users and other hospital information management systems. Authorized users can access the Web application from their secure hospital network computer by using the hospital’s browser technology and entering their username and password. The database holds up to 18 months of data and has the ability for each hospital to automatically backup data on a daily basis.

### Study Procedures

Participating hospitals transferred a database copy containing up to 18 months of infusion pump data to the investigator (April 2014 to February 2017) for periodic analytic services. Data was imported and sent via secure file transfer protocol (SFTP) to a secure central server protected by firewalls that are Health Insurance Portability and Accountability Act (HIPAA) deidentified (as necessary), and merged with a dedicated, secure data repository. A subset of these data containing a consecutive 60-day timeframe (ie, same quantity of days) for each hospital will be used in the statistical analyses.

The infusion pump has 11 types of alarms. A listing of alarms, associated cause(s), and effect(s) can be found in Table 1. All of the alarms produce an audible sound and visual signal on the pump screen indicating the type of alarm. All of the alarms are continuous and require clinician intervention to silence/resolve. Most of the alarms will stop the infusion or prevent it from being initiated until the alarm condition is resolved by the clinician.

#### Inclusion criteria

1. Hospitals that use one particular model pump (B. Braun Outlook 400ES Safety Infusion System) with the accompanying infusion management software (DoseTrac) and have agreed to the terms of a signed data licensing agreement, which allows the investigator access to infusion management software database data.
2. Pumps and infusion management software database data alarm records must include complete and consistent data elements to be included in the analysis. A complete alarm record will include all matching and consistent data elements from alarm start to alarm silence.

#### Exclusion criteria

1. Alarm records with potentially missing data resulting in incomplete records will be excluded. This includes records where an alarm is initiated with no accompanying end record, indicated by a hold state to silence and address the alarm, and any alarm record with data elements that differ at the start of the alarm from the end of the alarm (eg, drug name, concentration, rate, dose, VTBD, KVO volume does not equal zero, or different version of drug library) [23].
2. Alarm records associated with duplicate infusion pump serial numbers of “0” will be excluded from analyses. Infusion pump serial numbers can revert to “0” after major biomedical repairs. If the original pump serial number is not reentered, a hospital might have multiple pumps with a “0” serial number. As a result, the data from these “0” serial number pumps would appear to originate from a single device, while in actuality could result from a combination of data elements from multiple devices [23].

### Data Collection

Each hospital periodically copies and transfers a back-up of software database data (containing up to 18 months of data) via SFTP to a central server for requested analytic services. Patient Personal Health Information is deidentified prior to inclusion into a secure data repository. Access to the secure database is secured by Citrix and Virtual Private Network passwords and is accessible by a single Information Technology (IT) administrator/investigator. The database is backed-up daily for both local and off-site storage. The IT administrator/investigator who manages the central data repository does not interpret the data.

**Table 1:** Infusion pump alarm types, associated cause(s), and effect(s).

Alarm Type	Cause	Effect
Air In Line	Air-in-line sensor at pump detects air in the intravenous (IV) tubing (eg, due to improper priming or venting of tubing, collection of micro bubbles, negative pressure and out gassing, residual air in container)	Pump infusion stops and alarms until user resolves
Bag Empty	Empty container; pump cannot pull from container	Pump infusion stops and alarms until resolves
Battery Empty	Battery power is nearly fully discharged with approximately 3 minutes remaining	Pump alarms until plugged into outlet or, if it reaches fully discharged battery state, the pump powers off and the infusion is stopped
Check Set	IV tubing improperly loaded into pump; free flow clip may not be fully engaged	Pump alarms and infusion cannot be initiated until user resolves
Door Open	Door is opened while infusing; pump was not put in hold prior to opening door	Pump infusion stops and alarms until user resolves
Downstream Occlusion	IV line is occluded below the pump (eg, lower roller clamped, tubing kinked, or IV catheter occluded)	Pump infusion stops and alarms until user resolves
Hold Time Exceeded	Hold time has been exceeded; user put pump on hold and did not restart infusion	Pump remains in hold state (not infusing) and alarms until user resolves
KVO (Keep Vein Open)	Programmed volume to be delivered (VTBD) has infused (has reached 0 mL)	Pump infusion rate decreases to 3 mL/hour (or if programmed rate is <3 mL/hour it will continue to infuse at same rate) and alarms until user resolves (put infusion on hold and program new VTBD)
Low Flow From Container	IV line is partially occluded above the pump (eg, upper roller clamp partially closed or tubing kinked)	Pump infusion stops and alarms until user resolves
System Error	Device requires reboot or service	Pump alarms (not infusing) and continues until powered off. If reboot (power cycle) does not resolve, then user must send the pump to biomedical engineering for service
Upstream Occlusion	IV line is occluded above the pump (eg, upper roller clamp closed or tubing kinked)	Pump infusion stops and alarms until user resolves

Since hospital alarm data are not currently preassembled into a standardized interface report on this version of software, it will be necessary to collate and assemble the data in a meaningful way, using a data assembly tool. Collating data across multiple hospitals and running queries on that data entails intricate manipulation of enormous amounts of complex data. Microsoft SQL Server (2012 - 11.0.5343.0 X64), a database that supports a mix of transaction processing, data warehousing, and analytics applications, will be used to help manage the data. Unique code will be written to sort the data to complete the descriptive analyses (percent, mean, median, mode, range, and frequencies, as appropriate). In addition, anticipating that there might be clinically nonsignificant alarm data in the database, a data cleaning process has been developed and tested to detect, diagnose, and edit out clinically nonsignificant data. The methodology to be used in this study for collating, cleaning, and assembling valid and reliable pump alarm data across multiple hospitals was previously reported [23].

Pump data are complex and potentially contain data falling within certain preidentified data exclusions. Only data that does not fall within the exclusion principles will be included in the analysis. Additionally, elements of the software management database data need to be combined to ensure capture of the particular information needed to answer our research questions. Individual hospital names will be visible only to investigators, as this is relevant to collating and comparing hospital metrics (eg, census, staffed beds, case mix index), but will not be published.

### Data Elements to be Analyzed

A subset of data collected between April 2014 and February 2017, which encompasses a consecutive/concurrent 60-day interval, will be selected from each hospital backup database and then analyzed as a combined group. If this is not possible, a consecutive 60-day timeframe will be used.­­ Data elements to be analyzed from the software management database are listed in Textbox 1.

### Planned Statistical Analysis

The exact sample size for each data point/element was not statistically determined. The study will be exploratory in nature, thus the complete census of hospitals/pumps/alarm occurrences (minus those meeting the exclusion criteria) will be analyzed. Specific findings will be reported using the aggregate data for each hospital by care area, drug infused, time of day, and day of week, including: overall infusion pump alarm frequency (number of alarms per active infusion), duration of alarms (average, range, median), and type of alarms that occur in each hospital (percent breakdown of each type of alarm for each hospital). All data will be summarized and displayed in tables using descriptive statistics including mean, standard deviation, median, minimum, and maximum. Plots/graphs may also be used to summarize and display these data.

**Data elements that will be analyzed from the software management database. KVO: Keep Vein Open.**

**Facility Information**

- Facility name
- Number of hospital sites
- Number of pumps purchased
- Number of pumps in use
- Pump “go-live” date
- Number of licensed/staffed beds
- Number of annual admissions
- Case Mix Index

**Data Per Alarm**

- Date and time stamp (start and stop of alarm)
- Pump serial number
- Care Area (selected within drug library)
- Location (if this feature used, unit or department name)
- Mode (eg, primary, secondary, basic, drug library)
- State (alarm, hold, run, KVO)
- Drug name
- Alarm type

**Metrics**

- Active delivery state: total time (minutes:seconds) and percentage of time pumps in hold, run, alarm, and KVO state; total time from “run” to “off” that a pump can alarm
- KVO state: total time (minutes:seconds) and percentage of time pumps in KVO state
- Alarm state: total time (minutes:seconds) and percentage of time pumps in alarm and KVO state
- Alarm frequency by type: number of alarms that fall within each alarm type in the defined date range
- Total deliveries: number of unique medication deliveries running in the defined date range
- Average alarms per delivery: total number of alarms/total deliveries
- Average alarm duration: cumulative duration (minutes:seconds) of all alarm states/total number of alarms
- Total pumps available for analyses: number of unique pump serial numbers recorded in the defined date range

One-way analyses of variance (ANOVAs) will be used to determine any statistically significant differences between the hospitals, care areas, drugs, time of day, and day of week. Level of significance will be set at *P*<0.01. If statistically significant overall differences are found between the various groups, appropriate post hoc tests will be conducted to determine specifically where the differences exist. If the data meet the assumption of homogeneity of variances, Tukey's honestly significant difference post hoc test will be used. If the data do not meet the homogeneity of variances assumption, the Games Howell post hoc test will be used.

Microsoft SQL Server (2012 - 11.0.5343.0 X64), a database designed to support a mix of transaction processing, data warehousing, and analytics applications, will be used to manage the data and unique code will be written to collate and assemble the data to complete descriptive analyses. Specific applicable files will be transferred to Statistical Package for the Social Sciences Statistics version 22 (IBM-SPSS Inc, Armonk, NY) to complete the significance testing.

### Ethical Considerations

This study will be conducted in full accordance with all applicable Federal and State laws and regulations, including 45 CFR 46 and the HIPAA Privacy Rule. Each hospital’s alarm data will be blinded and cleaned prior to analysis. All patient-identifying data will be removed prior to merging into the dedicated and secure data repository.

To protect the integrity and security of the data, the infusion pump only uses one-way ports, meaning that data is transmitted only from the pump to external applications (eg, data management software). Data cannot be received by these outbound ports. The data management software port is also one-way, receiving inbound data only with a specific format, including a prefix, data, and checksum. Invalid incoming messages are rejected. The pump also utilizes a specific set of encryption/authentication schemes to meet hospital network setting requirements. The outbound infusion data format is industry standard Health Level-7 messaging and Integrating the Healthcare Enterprise - Patient Care Device compliant. Infusion data are transmitted over the hospital network to the infusion management software. As outlined in the infusion management software schedule and software terms of use (internal documents), investigators have been authorized to use certain data stored by the software on a deidentified, aggregate basis. Institutional Review Board approval for this work was not sought as the study will not directly involve human participants.

## Discussion

### Potential Benefits to Patients

Alarms from all medical devices are either nonactionable or actionable. Alarms emanating primarily from physiological monitoring devices have a higher proportion of being nonactionable while infusion pump alarms are mostly actionable. Nonactionable alarms have been associated with increased clinical staff desensitization toward all types of alarms. The resulting alarm fatigue has been identified as a critical safety issue resulting in potentially dangerous delays or nonresponse to actionable alarms contributing to serious patient injury and death. To date, there have been very limited analyses to show how infusion pump alarms specifically contribute to alarm fatigue and impact patient care. If we can better determine the incidences and characteristics of infusion pump alarms we can begin to focus on potential pump and clinical workflow design changes that prioritize rapid clinician response time for the actionable alarms required to ensure patient safety.

This study is designed to provide new insights regarding the prevalence and characteristics of infusion pump alarms related to the types and frequency of these alarms, including: alarm type, duration, day of week, time of day, drug name, care area, location, number of deliveries, and number of pumps in use. The benchmarking of this data, in addition to the analysis of the consistencies and inconsistencies of alarm types and frequencies between hospitals and hospital units, will help lay the foundation for future research initiatives, ultimately resulting in a decrease in alarm fatigue and better patient care and outcomes.

### Strengths and Limitations

This study will be an important first step in benchmarking types and frequencies of infusion pump alarms. However, the resulting data analysis will be limited since it will only include datasets from hospitals that use one particular large volume pump and its associated infusion management software, and have agreed to participate in the study by signing a data licensing agreement that allows investigator access to their infusion source data. In addition, datasets will vary in size (number of months) and may vary based upon time of year (in the event the analysis of concurrent datasets is not possible). Analyzing the same total number of months (ie, last 60 days of data available for each hospital site) will result in a comprehensive alarm data set, but this analysis will not include variables that could impact results like equality in time of year, pumps per patient ratio, or patient acuity.

## Abbreviations

ECG: electrocardiogram
ECRI: Emergency Care Research Institute
HIPAA: Health Insurance Portability and Accountability Act
ICU: intensive care unit
IT: information technology
IV: intravenous
KVO: Keep Vein Open
SFTP: secure file transfer protocol
VTBD: volume to be delivered
